# Apolipoprotein E e4 allele status and later-life depression in the Lothian Birth Cohort 1936

**DOI:** 10.1017/S0033291721000623

**Published:** 2022-12

**Authors:** Matthew H. Iveson, Adele Taylor, Sarah E. Harris, Ian J. Deary, Andrew M. McIntosh

**Affiliations:** 1 Centre for Clinical Brain Sciences, The University of Edinburgh, Edinburgh, UK; 2 Mental Health Data Science Scotland, Edinburgh, UK; 3 Lothian Birth Cohorts, The University of Edinburgh, Edinburgh, UK

**Keywords:** Apolipoprotein, depression, epidemiology, later-life, life-course, longitudinal

## Abstract

**Background:**

Previous results have been mixed regarding the role of the apolipoprotein E e4 (*APOE* e4) allele in later-life depression: some studies note that carriers experience greater symptoms and increased risk while others find no such association. However, there are few prospective, population-based studies of the *APOE* e4-depression association and fewer that examine depressive symptom trajectory and depression risk longitudinally. We examined the association between *APOE* e4 allele status and longitudinal change in depressive symptoms and depression risk in later-life, over a 12-year follow-up period.

**Methods:**

We used data from 690 participants of the Lothian Birth Cohort 1936 who took part in the Scottish Mental Survey 1947 (aged 11) and were followed-up in later-life over five waves from 2004 to 2019 (aged 70–82). We used *APOE* e4 allele status to predict longitudinal change in depressive symptom scores and risk of depression (defined by a symptom score threshold or use of depression-related medication). Models were adjusted for sex, childhood cognitive ability, childhood social class, education, adult social class, smoking status and functional limitations at baseline.

**Results:**

Depressive symptom scores increased with age. Once adjusted for covariates, *APOE* e4 allele status did not significantly predict symptom score trajectories or depression risk. Greater functional limitations at baseline significantly predicted poorer symptom score trajectories and increased depression risk (defined by medications). *APOE* e4 allele status did not significantly moderate the contribution of sex, education or functional limitations.

**Conclusions:**

There was no evidence that *APOE* e4 carriers experience an increased risk for later-life depression.

## Introduction

The apolipoprotein E e4 (*APOE* e4) allele plays an important role in the onset of dementia (Corder et al., 1993; Verghese, Castellano, & Holtzman, 2011) and the cognitive decline commonly observed across later-life (Davies et al., 2015, 2014; Deary et al., 2002; Luciano et al., 2009). In particular, the *APOE* e4 allele is associated with the impaired maintenance of myelin (Bartzokis et al., 2006) and beta-amyloid metabolism (Morris et al., 2010). However, *APOE* e4 is notably pleiotropic (Tuminello & Han, 2011), and has been implicated in several other later-life outcomes such as increased cardiovascular risk (Song, Stampfer, & Liu, 2004).

However, there is growing evidence that *APOE* e4 is particularly important for later-life depression. For example, in a meta-analysis of primarily candidate gene studies, Tsang and colleagues reported that *APOE* e4 carriers had significantly higher odds of later-life depression (clinical diagnosis or high symptom scores) (Tsang, Mather, Sachdev, & Reppermund, 2017), although this was only relative to e3 allele carriers and was largely driven by a single candidate gene study, with several other reviewed studies reporting no significant association. Despite this, there have been relatively few attempts to examine the role of *APOE* e4 status in depression risk prospectively and among population-based samples. The few existing studies have produced mixed findings, with some finding that *APOE* e4 carriers have greater depressive symptoms and higher risk of later-life depression (Skoog et al., 2015) and others finding less substantial (Burns, Andrews, Cherbuin, & Anstey, 2020) or non-significant (Locke et al., 2013; Tully, Péres, Berr, & Tzourio, 2016) associations. *APOE* was also not identified as a predictor of depression in two recent genome-wide association studies (Howard et al., 2019; Wray et al., 2018).

In a recent study, Burns and colleagues examined associations between *APOE* e4 status and depression over 12-year follow-up, both in terms of symptom scores and depression risk (identified by Brief Patient Health Questionnaire and Goldberg Depression Scale cut-offs) (Goldberg, Bridges, Duncan-Jones, & Grayson, 1988; Kroenke, Spitzer, & Williams, 2001), among participants of the Personality and Total Health (PATH) Through Life study (Burns et al., 2020). In older adults aged 60–64 at baseline, Burns et al. noted that *APOE* e4 carriers experienced a significant although small increase in depression symptom scores over the study period *v.* non-e4 carriers. However, no corresponding increase was observed in the risk of depression.

Given the mixed evidence regarding *APOE* e4 as a risk factor for later-life depression, further research is needed to replicate these findings. The current study examines the association between *APOE* e4 status and both longitudinal change in depression symptom scores and depression risk in the Lothian Birth Cohort 1936 (LBC1936), a large and representative sample of older adults with over 12 years of longitudinal follow-up (Deary et al., 2007). We also examine the contribution of a range of covariates and potential confounders from across the life course – including early-life cognitive ability, socioeconomic circumstances and smoking status – that are considered risk factors for depression in later-life (Gilman, Kawachi, Fitzmaurice, & Buka, 2002; Lorant et al., 2007; Lyness et al., 1998; Verropoulou, Serafetinidou, & Tsimbos, 2019). Consistent with the most recent of previous study (Burns et al., 2020), we hypothesise that depression symptom scores will increase across later-life and that *APOE* e4 carriers will experience a significantly greater increase than non-e4 carriers, independently of covariates such as sex and childhood cognitive ability. In contrast, we hypothesise that there will be no significant difference in depression risk – approximated using symptom score or medication-related cut-offs – between e4 allele carriers and non-carriers.

## Methods and materials

### Sample

The sample consisted of members of the Lothian Birth Cohort 1936 (LBC1936). These individuals were recruited from surviving participants of the Scottish Mental Survey 1947 (The Scottish Council for Research in Education, 1949) – a nationwide assessment of cognitive ability given to almost all children attending a Scottish school on the 4th June 1947 – who mostly still lived in the Edinburgh City and surrounding Lothian area at study commencement. The creation and design of the LBC1936 study is described in more detail elsewhere (Deary et al., 2007; Deary, Gow, Pattie, & Starr, 2012; Taylor, Pattie, & Deary, 2018). The LBC1936 study is conducted with ethical approval from the Research Ethics Committee for Scotland (wave 1: MREC/01/0/56), the Lothian Research Ethics Committee (wave 1: LREC/2003/2/29) and the Scotland a Research Ethics Committee (waves 2–5: 07/MRE00/58), and in accordance with the Declaration of Helsinki. Participants provided written informed consent at each wave.

The current study used data from five waves of the LBC1936 study: wave 1 was collected in 2004–2007 (*N* = 1091, *M* age = 69.53 years), wave 2 was collected in 2007–2010 (*n* = 866, *M* age = 72.49 years), wave 3 was collected in 2011–2013 (*n* = 697, *M* age = 76.25 years), wave 4 was collected in 2014–2017 (*n* = 550, *M* age = 79.32 years) and wave 5 was collected in 2017–2019 (*n* = 431, *M* age = 82.01 years). Individuals took part in a median of four waves.

The analytic sample was formed according to the criteria described by Burns et al. (2020). Notably, all participants in the LBC1936 were white. We first removed individuals who did not have genotyping data (*n* = 63), then individuals with Hospital Anxiety and Depression Scale – Depression subscale scores of 8 or above at wave 1 (see below; *n* = 49), then individuals self-reporting stroke at any wave (*n* = 121), and then individuals with cognitive impairment at any wave (Mini Mental State Examination scores <27; *n* = 168) (Folstein, Folstein, & McHugh, 1975). This left an analytic sample of 690 individuals: wave 1: *N* = 690, wave 2: *N* = 529, wave 3: *N* = 411, wave 4: *N* = 320, wave 5: *N* = 261 (median waves = 3).

### Assessments

#### APOE

DNA was extracted from whole blood samples collected at wave 1. *APOE* alleles (e2, e3 or e4) were established by genotyping the rs7412 and rs429358 single nucleotide polymorphisms with TaqMan assays (Applied Biosystems, Carlsbad, CA, USA) at the Wellcome Trust Clinical Research Facility Genetics Core, Western General Hospital, Edinburgh. *APOE* genotype consists of any two of the e2, e3 or e4 alleles, and has shown to be in Hardy–Weinberg equilibrium for the LBC1936 (*p* = 0.62) (Luciano et al., 2009). A binary variable was created to indicate the presence of an e4 allele.

#### Depression symptoms

At each of the five waves of follow-up, participants completed the Hospital Anxiety and Depression Scale (HADS) (Zigmond & Snaith, 1983), which asks participants about symptoms experienced over the previous week. Total scores from the depression subscale (scores’ range: 0–21) were used to measure depression symptoms, with higher scores indicating more severe symptoms.

#### Depression risk

Two binary indicators of depression status were created for each wave in order to measure depression risk. For the first indicator, depression was defined as a HADS – Depression subscale total score equal to or greater than 8. Previous study has shown this cut-off to have good specificity (0.79) and sensitivity (0.9) for depression (Bjelland, Dahl, Haug, & Neckelmann, 2002). For the second indicator, depression was defined as the presence of one or more keywords in the prescribed medications self-reported by each individual (see online Supplementary material for a full list of keywords). Individuals reported current medications as part of a structured interview conducted at each wave. Note that these indicator variables capture likely depression of any type, including mixed presentations with anxiety or psychotic symptoms, rather than pure depression.

#### Covariates

Covariates were chosen to align with those used by previous study (Burns et al., 2020), with the addition of variables commonly used in LBC1936 work. These covariates are commonly reported as risk factors for later-life depression (Burns et al., 2020; Gilman et al., 2002; Lorant et al., 2007; Lyness et al., 1998; Verropoulou et al., 2019).

Sex and self-reported years spent in full-time education were recorded at wave 1. Years of education was *z*-transformed prior to analysis.

Physical health was measured using total score (max possible = 18) on the Townsend Disability Scale (Townsend, 1962), completed at wave 1. Higher scores indicate more limited functional health. Due to positive skew, total scores were transformed using Tukey's Ladder of Powers (Tukey, 1977), raising scores to the power of 0.4.

Childhood cognitive ability was measured using the total score (max possible = 76) on the Moray House Test No. 12 (MHT), completed in 1947 as part of the Scottish Mental Survey 1947 (The Scottish Council for Research in Education, 1949). Scores were age-adjusted to account for the small differences in age at MHT, and then IQ-transformed for analysis (*M* = 100, s.d. = 15).

Childhood and adulthood socioeconomic positions were using measured using father's occupation and own highest occupation respectively, as retrospectively reported at wave 1. For women, husband's occupation was used where it was higher than their own. Occupations were coded into one of five occupation social classes (Professional, Managerial and Technical, Skilled, Partly-Skilled and Unskilled) according to the 1950 United Kingdom's classification index (General Register Office, 1956). The order of these classes was reversed so that higher-numbered classes represented more professional occupations.

Participants reported lifetime smoking experience at wave 1. A binary variable was created to indicate lifetime smoking status (ever smoked *v.* never smoked).

Death was measured with both a binary indicator variable (alive *v.* deceased) and age (in days) at death.

### Statistical analyses

The analyses were intended to closely resemble those of previous study (Burns et al., 2020).

Multilevel mixed effects models were constructed to examine the association between *APOE* e4 allele status and longitudinal change in depression symptom scores. Random intercepts and slopes were included for each participant. The univariate contribution of *APOE* e4 allele status was examined before adjusting for sex, years of full-time education (*z*-score), Townsend Disability Scale score (Tukey-transformed), age-adjusted MHT score (IQ-scaled), father's occupational social class (reversed), own occupational social class (reversed) and smoking status. Consistent with Burns et al., interaction effects were included between *APOE* e4 allele status and sex, years of full-time education and Townsend disability score (Burns et al., 2020).

Competing risks survival analyses were conducted to examine the association between *APOE* e4 allele status and the risk of depression across the follow-up period. In particular, we examined depression onset and death as competing risks; those individuals who die are no longer at risk of depression, and the *APOE* e4 allele has been associated with other health conditions that affect longevity (Ewbank, 2004; Song et al., 2004). Separate analyses were conducted using depression status derived from either HADS-D clinical cut-off (scores ≥8) or from reported medications (depression-related medications; see online Supplementary material). In both analyses, depression was not treated as a recurrent event, as the wave-based measurement of depression makes it difficult to identify the start and end of depressive episodes. As with the mixed effects analysis, the univariate contribution of *APOE* e4 allele status was examined before adjusting for covariates. No interaction effects or time-dependent covariates were included. The proportional hazards assumption was met for *APOE* e4 allele status (*p* = 0.18) and for all included covariates (*p*s > 0.10).

All analyses were conducted in R (R Core Team, 2018) using RStudio (RStudio Team, 2016), and with the ‘lme4’ (Bates, Mächler, Bolker, & Walker, 2015), ‘psych’ (Revelle, 2018), ‘survival’ (Therneau & Lumley, 2015) and ‘cmprsk’ (Gray, 2019) packages. Models were not adjusted for multiple comparisons. In order to assess whether results were partly driven by the specific categorisation of *APOE* e4 status, we additionally conducted sensitivity analyses in which the mixed effects and competing risks analyses were repeated using a binary *APOE* e4 status variable that contrasts e4 allele carriers (e4/e4 and e3/e4) with e3/e3 individuals, removing e2 carriers (online Supplementary material).

## Results

### Sample descriptives

**Fig. 1.**
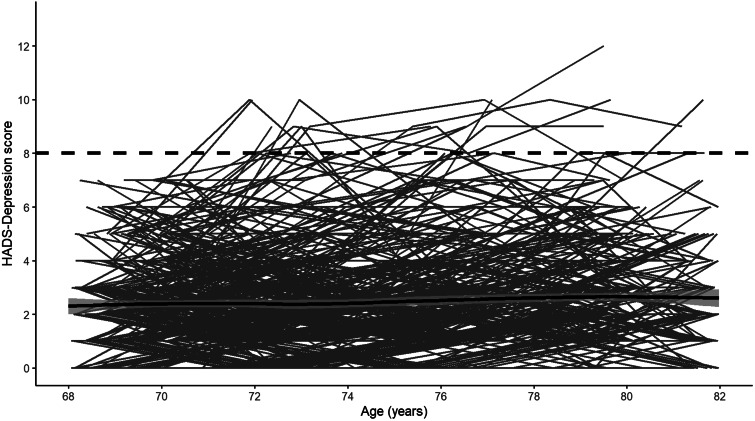
Individual longitudinal trajectories of HADS-Depression scores. The solid black line shows the mean trajectory (smoothed loess regression) with 95% confidence intervals and the dashed black line shows the cut-off used. *Note*: The analytic sample only includes individuals with HADS-Depression scores <8 at baseline. Waves 1–5 were measured at mean ages of 70, 72, 76, 79 and 83 years-old.

Descriptive statistics for the analytic sample are shown in Table 1. Compared to removed individuals, individuals included in the analytic sample were significantly more likely to be female (*p* < 0.01), report more years of education (*p* < 0.01), be from higher occupational social classes (*p* < 0.001), report less functional impairment (*p* = 0.003), report fewer depressive symptoms at each wave (wave 1: *p* < 0.001; wave 2: *p* < 0.001; wave 3: *p* < 0.001; wave 4: *p* = 0.01; wave 5: *p* < 0.001; Fig. 1) and be below the depression cut-off (HADS Depression score *p* < 0.001; medications *p* = 0.02). Notably, individuals in the analytic sample did not significantly differ from the removed individuals in terms of *APOE* genotype distribution (*p* = 0.08) or vitality status (*p* = 0.62).

**Table 1.** Descriptives for the analytic sample (*N* = 690)

	*N* (%)	Mean (s.d.)	*N* missing (%)	Comparison with removed individuals *p*
Sex			0 (0%)	<0.01
Male	323 (47%)			
Female	367 (53%)			
Full-time education (years)			0 (0%)	<0.01
Mean (s.d.)		10.82 (1.14)		
Father's social class			66 (10%)	0.54
Unskilled	49 (7%)			
Partly skilled	53 (8%)			
Skilled	345 (50%)			
Intermediate	132 (19%)			
Professional	45 (6%)			
Own social class			9 (1%)	<0.001
Unskilled	0 (0%)			
Partly skilled	21 (3%)			
Skilled	267 (39%)			
Intermediate	257 (37%)			
Professional	136 (20%)			
Moray House Test score (age-corrected)			30 (4%)	0.22
Mean (s.d.)		48.94 (2.26)		
Townsend Disability Scale score			1 (0%)	<0.01
Mean (s.d.)		0.84 (1.72)		
Smoking status			0 (0%)	0.10
Never smoked	330 (48%)			
Ever smoked	360 (52%)			
*APOE* genotype			0 (0%)	0.08
e2/e2	1 (0%)			
e2/e3	87 (13%)			
e2/e4	16 (2%)			
e3/e3	406 (59%)			
e3/e4	169 (24%)			
e4/e4	11 (2%)			
HADS – Depression score wave 1			0 (0%)	<0.001
Mean (s.d.)		2.40 (1.71)		
HADS – Depression score wave 2			162 (23%)	<0.001
Mean (s.d.)		2.36 (1.95)		
HADS – Depression score wave 3			279 (40%)	<0.001
Mean (s.d.)		2.52 (2.06)		
HADS – Depression score wave 4			370 (54%)	0.01
Mean (s.d.)		2.68 (1.97)		
HADS – Depression score wave 5			430 (62%)	<0.001
Mean (s.d.)		2.67 (2.03)		
Depressed status – HADS cut-off			0 (0%)	<0.001
Never depressed	656 (95%)			
Ever depressed	34 (5%)			
Depressed status – Medication cut-off			0 (0%)	0.02
Never depressed	624 (90%)			
Ever depressed	66 (10%)			
Deceased status			0 (0%)	0.62
Alive	473 (69%)			
Deceased	217 (31%)			

*p* indicates comparison between the analytic sample (*N* = 690) and removed individuals (*N* = 401); Kruskal–Wallis tests are used for numeric variables and χ^2^ tests are used for categorical variables.

The analytic sample was predominantly female, well-educated and reported few functional limitations. Roughly, a quarter (*N* = 180) of the analytic sample possessed an *APOE* e4 allele; the majority of individuals possessed the e3/e3 genotype. The distribution of *APOE* genotypes was in Hardy–Weinberg equilibrium (χ^2^ = 9.76, *p* = 0.08). There was no evidence of differential dropout by *APOE* e4 status among the analytic sample after wave 1, with no significant differences in the distribution of *APOE* genotypes (wave 2: *p* = 0.61; wave 3: *p* = 0.75; wave 4: *p* = 0.73; wave 5: *p* = 0.52) or in the number of e4 alleles (wave 2: *p* = 0.98; wave 3: *p* = 0.44; wave 4: *p* = 0.35; wave 5: *p* = 0.08) between those present or absent at each wave. Furthermore, number of waves completed was not significantly associated with APOE genotype (*p* = 0.63) or number of e4 alleles (*p* = 0.36). On average, very few depressive symptoms were reported at each wave, although mean depression symptom scores increased slightly with time. However, few individuals met either the symptom score cut-off (HADS Depression scores ≥8; 5%) or the medication cut-off (any depression-related prescription; 10%) across the five-wave follow-up. In terms of overlap, eight individuals met both cut-offs, 26 individuals met only the symptom score cut-off (not the medication cut-off), 58 individuals met only the medication cut-off (not the symptom score cut-off) and 598 individuals met neither cut-off.

### *APOE* e4 and depression symptom scores

Visual inspection of the averaged HADS Depression score trajectory, smoothed using Loess regression, suggested a linear increase in scores over time (Fig. 1). Indeed, HADS Depression scores were significantly predicted by wave (*β* = 0.04, s.e. = 0.01, *p* < 0.001) in a linear growth model. Although scores were also significantly predicted by both a linear (*β* = 3.95, s.e. = 0.98, *p* < 0.001) and a quadratic effect (*β* = 2.02, s.e. = 0.93, *p* = 0.03) of wave in an orthogonal quadratic growth model, this model did not significantly improve model fit relative to the linear growth model [linear growth Akaike's information criteria (AIC) = 7915.20, quadratic growth AIC = 7915.20, χ = 8.04, df = 4, *p* = 0.09]. We therefore modelled growth in HADS Depression scores as linear in further analyses. In the univariate model, the *APOE* e4 allele was not significantly associated with longitudinal change in HADS Depression scores (Table 2). In the fully-adjusted model (Table 2), greater functional limitations at baseline were significantly associated with an increase in depressive symptomology over time. No other covariate, including interaction effects, significantly predicted longitudinal change in HADS Depression scores. A similar pattern of associations was observed when using the e4 *v.* e3/e3 binary variable (online Supplementary material).

**Table 2.** Relationship between *APOE* e4 allele status – univariate and adjusted for covariates – and both longitudinal change in depressive symptom scores (multilevel mixed effects regression) and depression risk (HADS – Depression cut-off, Medication cut-off and combined; competing risks regression)

	HADS-Depression scores		Depression risk – HADS cut-off		Depression risk - Medication cut-off		Depression risk – Combined	
	IRR (95% CI)	*p*	OR (95% CI)	*p*	OR (95% CI)	*p*	OR (95% CI)	*p*
Univariate model								
*APOE* e4 allele status – e4 allele	0.98 (0.88–1.10)	0.76	0.35 (0.12–0.98)	0.05	1.15 (0.68–1.95)	0.60	0.88 (0.55–1.40)	0.58
Multivariate model								
*APOE* e4 allele status – e4 allele	1.03 (0.86–1.23)	0.75	0.50 (0.17–1.48)	0.21	1.17 (0.63–2.16)	0.62	0.97 (0.56–1.66)	0.91
Sex – female	0.99 (0.87–1.13)	0.92	1.92 (0.80–4.65)	0.15	2.14 (1.16–3.96)	0.02	1.93 (1.15–3.24)	0.01
Years of full-time education (*z*-scaled)	0.99 (0.92–1.06)	0.79	1.05 (0.60–1.83)	0.88	1.14 (0.82–1.58)	0.44	1.12 (0.84–1.50)	0.44
Townsend Disability Scale score (Tukey-transformed)	1.38 (1.25–1.52)	<0.001	1.43 (0.86–2.40)	0.17	1.76 (1.21–2.54)	<0.01	1.70 (1.25–2.32)	<0.001
Age-adjusted MHT score (IQ-scaled)	1.00 (0.99–1.01)	0.91	1.00 (0.97–1.02)	0.76	0.98 (0.97–1.00)	0.11	0.99 (0.97–1.01)	0.20
Father's occupational social class (reversed)	1.00 (0.95–1.07)	0.88	0.85 (0.57–1.27)	0.42	0.92 (0.67–1.27)	0.63	0.88 (0.68–1.13)	0.31
Own occupational social class (reversed)	0.94 (0.86–1.02)	0.15	0.69 (0.42–1.13)	0.14	0.92 (0.60–1.40)	0.69	0.88 (0.62–1.25)	0.48
Lifetime smoking status – ever smoked	1.02 (0.91–1.13)	0.76	0.55 (0.25–1.20)	0.13	1.45 (0.84–2.51)	0.19	1.04 (0.65–1.65)	0.87
*APOE* e4 × Sex	0.97 (0.76–1.23)	0.77	–	–	–	–	–	–
*APOE* e4 × Education	1.03 (0.92–1.16)	0.60	–	–	–	–	–	–
*APOE* e4 × Townsend	0.91 (0.76–1.09)	0.32	–	–	–	–	–	–

IRR, incidence rate ratio; OR, odds ratio; HADS, Hospital Anxiety and Depression Scale; MHT, Moray House Test No. 12.

### *APOE* e4 and depression risk

**Fig. 2.**
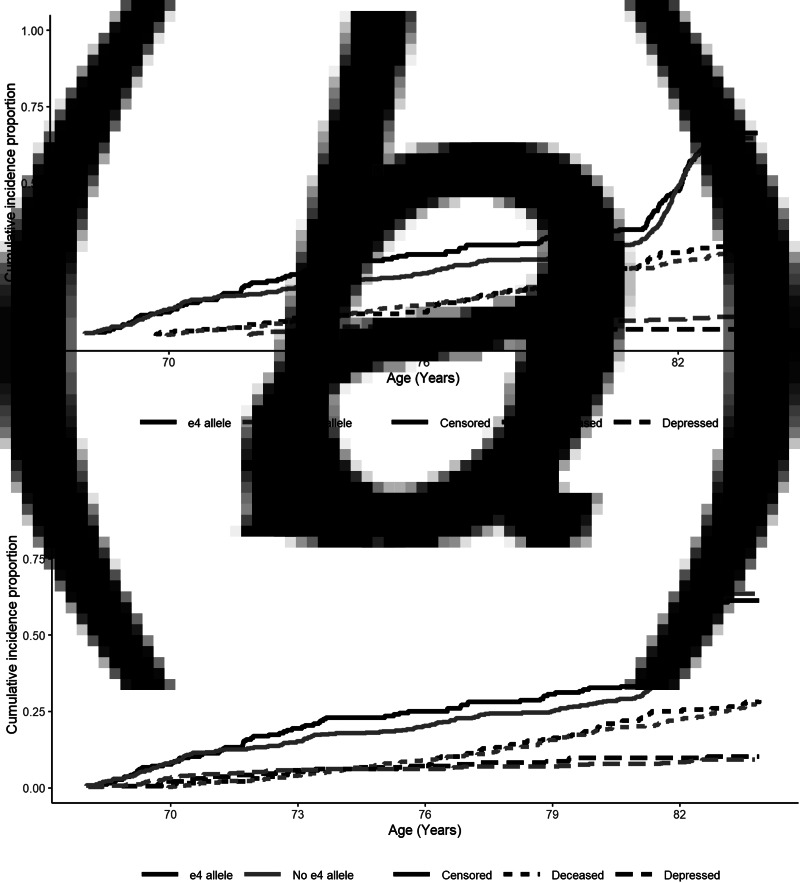
Cumulative risk curves for depression (dashed lines), deceased (dotted lines) and censored (attrited or survived to end of follow-up without being recorded as deceased or depressed; solid lines), split by *APOE* e4 allele status (e4 allele = black, no e4 allele = grey): (a) HADS-Depression score cut-off and (b) medication cut-off. *Note*: At baseline participants did not report depression according to the HADS-Depression score cut-off. Waves 1–5 were measured at mean ages of 70, 72, 76, 79 and 83 years-old. Depression and censored status were assessed at each wave, whereas deceased status was assessed continuously.

When examining depression risk derived from the HADS Depression score cut-off, individuals with the *APOE* e4 allele were marginally less likely to experience depression over the follow-up period (Table 2; Fig. 2a). However, this association did not appear to be robust, and was attenuated in the fully-adjusted model, where there were no significant predictors of depression risk (Table 2). This pattern of results was similar when using the e4 *v.* e3/e3 binary variable (online Supplementary material).

When examining depression risk derived from the medication cut-off, individuals with the *APOE* e4 allele were not at significantly increased risk across the follow-up period (Table 2). In the fully-adjusted model, being female and greater functional limitations were the only variables to significantly predict higher risk of depression (Table 2). In sensitivity analysis, using the adjusted e4 status variable, only greater functional limitations significantly predicted depression risk (online Supplementary material).

We additionally examined depression risk derived from a combination of the HADS Depression score and medication cut-offs, with the earliest identified episode used to estimate survival time. Individuals with the *APOE* e4 allele were not at significantly increased risk of depression over the follow-up period in either the univariate or fully-adjusted models (Table 2). Increased risk of depression in the fully-adjusted model was significantly predicted by being female and by greater functional limitations (Table 2). This pattern of results was similar when using the e4 *v.* e3/e3 binary variable (online Supplementary material).

## Discussion

The current study aimed to add to the few longitudinal population-based studies of the role of *APOE* in later-life depression and to extend previous study using a large, well-phenotyped birth cohort.

The current study found no significant association between the *APOE* e4 allele and longitudinal change in depressive symptoms or longitudinal risk of depression, even when directly contrasting e4 carriers to e3/e3 individuals (online Supplementary material). These findings conflict with those from a Swedish population-based sample of older adults (Skoog et al., 2015), although notably the follow-up in the current study was more frequent. These findings are also partly in conflict with those from the PATH Through Life study, in which older adults with the *APOE* e4 allele experienced a greater longitudinal increase in depressive symptoms than those without the *APOE* e4 allele (incidence rate ratio = 1.130) (Burns et al., 2020). However, due to the low number of symptoms reported, Burns et al. concluded that this increase among older adult *APOE* e4 carriers was not substantive or clinically significant. The non-significant result observed here supports this interpretation (see also Locke et al., 2013; Tully et al., 2016), although it is worth noting the smaller analytic sample here (*N* = 690) *v.* the over 60s sample in Burns et al. (*N* = 1768) (Burns et al., 2020). Consistent with recent longitudinal studies (Burns et al., 2020; Locke et al., 2013) and with recent large genome-wide association studies of broad depression phenotypes using predominantly middle-aged adults (Howard et al., 2019; Wray et al., 2018), the current study observed no significant association between the *APOE* e4 allele and depression risk once life-course covariates were accounted for.

The current study did find that HADS depression symptom trajectories were significantly predicted by Townsend disability scores around age 70; those with more functional limitations at baseline experienced greater increases in depression symptom scores over time. This is consistent with study showing that functional limitations place an increasing burden on mental health (Yang & George, 2005). Notably, functional limitations did not appear to predict risk of later-life depression using the HADS-Depression threshold, suggesting that the increase in symptoms remained below clinical thresholds. We did not, however, account for functional limitations as a time-dependent covariate or examine concurrent changes in disability and depression symptoms. It is possible that longitudinal (post-baseline) changes in functional health may also contribute to depressive symptom trajectories (Schieman & Plickert, 2007; Yang & George, 2005). Although more functional limitations did predict increased risk of depression measured by medication-use, several of the medications included in the definition may be prescribed for stress and pain, and so there is likely confounding with the limiting condition itself.

The current study also observed that, although depressive symptoms generally worsened with increasing age, overall scores were relatively low. This is consistent with several previous studies demonstrating that older adults generally exhibit fewer depressive symptoms than younger or middle-aged adults (Burns et al., 2020; Girling et al., 1995; Locke et al., 2013; Mauricio et al., 2000; Skoog et al., 2015; Varma, 2012).

### Limitations

Although we examined both longitudinal change in self-reported depressive symptoms and proxy indicators of depression, clinical diagnoses of depression were not available. The proxy indicators were created to capture any depression, including both pure presentations of depression (i.e. those reporting only depressive symptoms or only depression-related medications) and mixed presentations with additional anxiety, psychosis or apathy. Furthermore, we make no distinction between minor (i.e. where symptoms are not sufficiently severe or are too few to warrant a diagnosis of major depression) or major depression or between acute and chronic depression. As clinically distinct conditions, future research may benefit from examining the potentially differential role of *APOE* genotype in different presentations of depression, particularly given that previous study has suggested that *APOE* e4 confers a greater risk of minor but not major depression (Skoog et al., 2015).

Depression was not treated as a recurrent event in the analyses, in part because it was measured in waves rather than continuously. As there was roughly a 3-year gap between each wave, it is possible that individuals may have experienced depressive episodes in between waves. Such episodes would not be captured by the HADS questionnaire, which asks participants to rate how they felt over the preceding week only, or by prescribed medication self-reported at interview. Drop-out between waves may also have indicated attrition of the most depressed individuals or those most at risk for depression in later-life, resulting in an underestimation of depression risk and symptom change. Note, however, that depressive symptom scores increased slightly over the study period, and that presence of the *APOE* e4 allele did not predict death as a competing risk (HADS-Depression cut-off: *p* = 0.140; medication cut-off: *p* = 0.520).

Finally, in terms of the sample, there were relatively few individuals identified as ‘depressed’ at any point in the follow-up period using the HADS-Depression cut-off (*N* = 34; 5%). Such a low frequency may be partly explained by the narrow time window (previous 1 week) assessed by the HADS questionnaire. Although the medication cut-off did identify more individuals as ‘depressed’ (*N* = 66; 10%) – more consistent with the rates reported by previous study (11% in Burns et al., 2020) – individuals did not provide information about why these medications were prescribed or at what dosage. Furthermore, those classified as ‘depressed’ according to medications could have treated (i.e. not incident) depression, whereas the HADS questionnaire included only incident depression. The medication cut-off may therefore be too sensitive, and the corresponding results interpreted with caution. Note that *APOE* e4 allele status did not significantly predict depression risk using the medication cut-off.

### Strengths

The current study represents one of the first attempts to examine the role of *APOE* in later-life depression longitudinally among a single-year birth cohort. Where previous studies have used age-binned cohorts (e.g. 60s), significant associations may arise due to the confounding effect of age. In addition, the LBC1936 is one of the most extensively phenotyped birth cohorts, and provides the opportunity to account for a variety of important covariates from across the life course (Deary et al., 2007). For example, here functional limitations in older age were associated with greater increases in depressive symptomology and increased risk of later-life depression. The current study also represents the longest follow-up of older adults to date, with LBC1936 individuals followed for over 12-years.

## Conclusion

The current study is one of the largest longitudinal assessments of the role of *APOE* e4 in later-life depression symptomology and risk. We found no evidence for a greater increase in depressive symptoms or a greater risk of depression among *APOE* e4 allele carriers. This supports previous suggestions that older *APOE* e4 allele carriers are not at an increased risk for later-life depression.

## Supplementary material

For supplementary material accompanying this paper visit https://doi.org/10.1017/S0033291721000623. click here to view supplementary material
