# Supplementary material for: Apolipoprotein E e4 allele status and later-life depression in the Lothian Birth Cohort 1936
Source: Psychol Med. 2021 Mar 2;52(16):3816–24. doi: 10.1017/S0033291721000623 (PMC9811345; doi:10.1017/S0033291721000623)
Supplement: Supplementary file 1 [file S0033291721000623sup001.docx]

**Supplementary Material**

Depression-related medications

At each of the 5 waves of follow-up, participants self-reported any prescribed medications they were using. These free-text medication fields were used to create a depression indicator for each wave, with depression defined as the presence of one or more of the keywords presented in Table S1.

**Table S1.** Keywords used to identify depression from self-reported medications at each wave.

| **General depression-related terms** | depress; SSRI; serotonin; maoi; monoamine; tricyclic |
| --- | --- |
| **Seratonin Reuptake Inhibitors** | bupropion; citalopram; dapoxetine; duloxetine; escitalopram; fluoxetine; fluvoxamine; paroxetine; sertraline; venlafaxine; zyban; cipramil; priligy; dutor; yentreve; cymbalta; depalta; duciltia; cipralex; olena; prozep; faverin; seroxat; lustral; venlalic; sunveniz; venladex; viepax; majoven; vencarm; venlablue; alventa; depefex; efexor; politid; tonpular; venaxx; venlasov; vensir; venzip |
| **Tricyclics** | amitriptyline; clomipramine; dosulepin; doxepin; imipramine; lofepramine; mianserin; nortriptyline; trazodone; trimipramine; prothiaden; xepin; molipaxin |
| **MAOIs** | isocarboxazid; moclobemide; phenelzine; reboxetine; tranylcypromine; manerix; nardil; edronax |
| **Other depression-related medications** | buspirone; mirtazapine; naltrexone; reboxetine; tryptophan; vortioxetine; zispin; adepend; edronax; optimax; brintellix |

Cumulative incidence at specific ages

**Table S2.** Cumulative incidence functions for the competing risks of being depressed, deceased and censored at ages 72, 76, 79 and 83 (corresponding to median ages at Waves 2, 3, 4, and 5), grouped by *APOE* e4 allele status.

| **Outcome** | ***APOE* e4 status** | **Age 72** | | **Age 76** | | **Age 79** | | **Age 83** | |
| --- | --- | --- | --- | --- | --- | --- | --- | --- | --- |
|  |  | **Cumulative Incidence** | **Variance** | **Cumulative Incidence** | **Variance** | **Cumulative Incidence** | **Variance** | **Cumulative Incidence** | **Variance** |
| Depressed | No e4 allele | 0.01 |  | 0.03 |  | 0.05 |  | 0.06 |  |
|  | e4 allele | 0.00 |  | 0.00 |  | 0.02 |  | 0.02 |  |
| Deceased | No e4 allele | 0.03 |  | 0.10 |  | 0.17 |  | 0.27 |  |
|  | e4 allele | 0.04 |  | 0.08 |  | 0.16 |  | 0.29 |  |
| Censored | No e4 allele | 0.14 |  | 0.20 |  | 0.25 |  | 0.64 |  |
|  | e4 allele | 0.17 |  | 0.27 |  | 0.32 |  | 0.66 |  |

Wave 1 Median age = 69 years, Wave 2 Median age = 72 years, Wave 3 Median age = 76 years, Wave 4 Median age = 79 years, Wave 5 Median age = 83 years.

APOE e4 vs e3/e3

In their meta-analysis, Tsang and colleagues (Tsang, Mather, Sachdev, and Reppermund, 2017) note that the *APOE* e4 allele was significantly associated with the risk of late-life depression only when contrasted with the e3 allele. Furthermore, they reported no significant association when contrasting the presence and absence of the e4 allele – the binary distinction used in the main text of the present study. It is therefore possible that the results reported in the main text were driven by the categorisation of the binary *APOE* e4 variable, and that the potential impact of the e4 allele is better assessed by contrasting it to the e3 allele. To test this, we repeated the mixed effects and survival analyses using a new binary *APOE* e4 variable, this time contrasting e3/e4 and e4/e4 carriers with e3/e3 carriers (N = 586). Note that this also involved removing those with an e2 allele from the analysis (N = 104). The result was a set of analyses that more directly addresses the e4 versus e3 comparison noted by Tsang et al. (2017).

The results from this sensitivity analysis are presented in Table S3, which can be compared to the e4+ versus e4- analyses presented in Table 2 of the main text. There were no notable differences between the two *APOE* allele groupings, with all IRRs and ORs of the same direction and of roughly the same size. As in the e4+ versus e4- analyses presented in the main text, higher disability was significantly associated with longitudinal increases in HADS Depression scores and with an increased risk of depression according to the medication cut-off. Furthermore, e4 carriers (versus e3 carriers) were at a marginally-significant increased risk of depression measured by the HADS Depression score cut-off, though this was not significant in the multivariate model once covariates were accounted for (see Table 2, Main Text). This suggests that the pattern of results reported in the main text is not driven by the categorisation of *APOE* e4 status used.

**Table S3.** Relationship between *APOE* e4 allele status (e3/e4 and e4/e4 vs e3/e3) – univariate and adjusted for covariates – and both longitudinal change in depressive symptom scores (multilevel mixed effects regression) and depression risk (HADS – Depression cut-off, Medication cut-off and combined; competing risks regression).

|  | **HADS-Depression scores** | | **Depression risk - HADS cut-off** | | **Depression risk - Medication cut-off** | | **Depression risk - Combined** | |
| --- | --- | --- | --- | --- | --- | --- | --- | --- |
|  | **IRR [95% CI]** | **p** | **OR [95% CI]** | **p** | **OR [95% CI]** | **p** | **OR [95% CI]** | **p** |
| **Univariate model** |  |  |  |  |  |  |  |  |
| *APOE* e4 allele status – e4 allele | 0.97 [0.86, 1.09] | 0.59 | 0.34 [0.12, 0.97] | 0.04 | 0.97 [0.54, 1.73] | 0.91 | 0.75 [0.45, 1.26] | 0.28 |
| **Multivariate model** |  |  |  |  |  |  |  |  |
| *APOE* e4 allele status – e4 allele | 1.04 [0.86, 1.25] | 0.69 | 0.49 [0.16, 1.49] | 0.21 | 0.95 [0.49, 1.85] | 0.88 | 0.82 [0.46, 1.47] | 0.51 |
| Sex – Female | 0.99 [0.86, 1.14] | 0.85 | 2.14 [0.87, 5.25] | 0.10 | 1.87 [0.98, 3.56] | 0.06 | 1.80 [1.05, 3.08] | 0.03 |
| Years of full-time education (z-scaled) | 1.00 [0.92, 1.09] | 0.99 | 1.03 [0.55, 1.91] | 0.94 | 1.01 [0.68, 1.50] | 0.97 | 1.02 [0.72, 1.43] | 0.92 |
| Townsend Disability Scale score (Tukey-transformed) | 1.42 [1.28, 1.57] | <0.001 | 1.50 [0.91, 2.47] | 0.12 | 1.74 [1.14, 2.67] | 0.01 | 1.74 [1.24, 2.44] | <0.01 |
| Age-adjusted MHT score (IQ-scaled) | 1.00 [1.00, 1.01] | 0.63 | 0.99 [0.96, 1.02] | 0.46 | 0.99 [0.97, 1.01] | 0.23 | 0.99 [0.97, 1.01] | 0.24 |
| Father’s Occupational Social Class (reversed) | 0.98 [0.91, 1.04] | 0.47 | 0.80 [0.53, 1.21] | 0.30 | 0.93 [0.64, 1.35] | 0.71 | 0.86 [0.65, 1.13] | 0.28 |
| Own Occupational Social Class (reversed) | 0.93 [0.85, 1.02] | 0.12 | 0.71 [0.43, 1.16] | 0.17 | 1.11 [0.72, 1.70] | 0.64 | 1.02 [0.71, 1.46] | 0.91 |
| Lifetime Smoking status – Ever smoked | 0.99 [0.88, 1.11] | 0.82 | 0.59 [0.26, 1.31] | 0.19 | 1.49 [0.81, 2.75] | 0.20 | 1.07 [0.65, 1.77] | 0.79 |
| *APOE* e4 X Sex | 0.97 [0.75, 1.25] | 0.83 | - | - | - | - | - | - |
| *APOE* e4 X Education | 1.02 [0.90, 1.15] | 0.80 | - | - | - | - | - | - |
| *APOE* e4 X Townsend | 0.88 [0.73, 1.07] | 0.19 | - | - | - | - | - | - |

IRR = Incidence Rate Ratio; OR = Odds Ratio; HADS = Hospital Anxiety and Depression Scale; MHT = Moray House Test No.12.

References

Tsang, R. S. M., Mather, K. A., Sachdev, P. S., & Reppermund, S. (2017). Systematic review and meta-analysis of genetic studies of late-life depression. *Neuroscience and Biobehavioral Reviews*, *75*, 129–139. https://doi.org/10.1016/j.neubiorev.2017.01.028
